# Consumer Acceptance of Novel Lucuma Fruit Ice Cream in the US Market

**DOI:** 10.3390/foods13193055

**Published:** 2024-09-25

**Authors:** Gaganpreet Singh, Rajesh Kumar, Martin J. Talavera

**Affiliations:** Sensory and Consumer Research Center, Department of Food, Nutrition, Dietetics, and Health, Kansas State University, Olathe, KS 66061, USA; gag4747@ksu.edu (G.S.); talavera@ksu.edu (M.J.T.)

**Keywords:** lucuma, ice cream, consumer, acceptance, sensory, food neophobia

## Abstract

This study explored the use of lucuma fruit powder in an ice cream formulation for the US market. Six ice cream prototypes were developed using five different lucuma fruit powder variants. A central location test was conducted with frequent ice cream consumers (*n* = 106) to assess acceptance, attribute intensity rating, ideal intensity levels, and purchase intent against a control sample with caramel flavor. The mean overall liking score for all lucuma ice creams was moderate. The overall, aroma, and flavor liking scores were significantly higher (*p* < 0.05) for lucuma ice cream samples, whereas the control sample was liked significantly more for texture. The Terrasoul variant of lucuma ice cream was the most liked among all samples, and the control was liked the least. Only the Terrasoul ice cream sample was successful in delivering significantly strong caramel, fruit, and sweet flavor levels; the other lucuma ice cream samples were more comparable to the control. The inclusion of lucuma powder increased powdery mouthfeel perception, negatively impacting texture liking. Consumers perceived all ice cream samples to be weak in flavor and fell short of delivering ideal levels. The US consumers had low–moderate food neophobia scores for lucuma fruit, with 57% showing interest in buying lucuma ice creams.

## 1. Introduction

The world is witnessing a growing demand among consumers for natural, plant-based, and healthy ingredients in food products. The key reasons are high nutritional value, low-calorie ingredients, natural sweetness, and new flavors [1]. Consumers intend to buy healthy products but do not want to compromise on taste or believe in changing their eating habits. Thus, food developers have been riding the wave of increasing health interest and have recognized the considerable potential of adding functional ingredients to regular products. Often, “superfood” is the term used for natural foods that are beneficial for human health because of richness in one or more nutrients [2]. Superfoods can consist of a variety of nutrients such as healthy fats, fiber, vitamins, and minerals, as well as offer health benefits and/or have properties of disease prevention [3]. Superfoods as ingredients are considered rich in components believed to reduce the onset of diseases such as cancer, diabetes, heart disease, etc., and can be considered both a food and a medicine [4].

**Figure 1:**
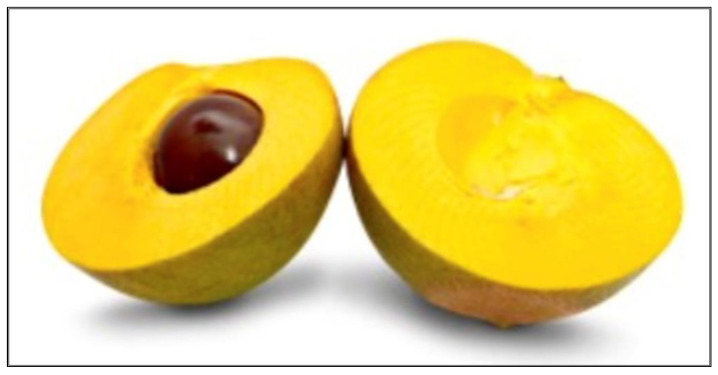
Lucuma concept presented to consumers before the purchase intent question. Note: Lucuma, also known as the “Gold of the Incas”, is a fruit native to Peru. It is high in beta-carotene, iron, zinc, calcium, protein, and fiber. It also contains antioxidants and potassium, which are said to be good for your heart, immune system, and skin. This fruit is used as a sweetener and as flavoring for beverages and desserts such as ice creams, custards, marmalades, and yogurt, among others.

Lucuma fruit is one of the many superfoods underutilized for its potential as an ingredient in food products. Lucuma (*Pouteria lucuma*) is a tropical fruit that belongs to the Sapotaceae family, mainly cultivated in the Andean region of Peru, Chile, and Ecuador. Peru is the largest producer (88% of worldwide production) and exporter worldwide [5]. Lucuma fruit has an ovoid to elliptical shape, is 7.5 to 10 cm in size, and the color of its skin varies from green to yellow, depending on the degree of ripeness. The flesh of the fruit is dry and starchy with an orange-to-yellow color (Figure 1). The fruit is highly sensitive and susceptible to physical injury and is usually commercialized as pulp and/or in powdered form [6]. The lucuma fruit is mainly processed into frozen fruit or pulp and powder/flour and then incorporated for use in food industries. Lucuma fruit powder has been used as an ingredient in the production of baby foods, pies, smoothies, chocolate, and yogurt [7,8], though, to date, its utilization has been limited to the Andean region.

Historically, lucuma fruit is also known as the “Gold of the Incas” and was used by the Inca civilization as one of the main ingredients of their diet [9]. Many studies have detailed the nutritional and health aspects of lucuma. Lucuma is known to be a good source of dietary fiber [10]. It has anti-inflammatory polyphenols and antioxidant compounds [11], flavonoid compounds and phenolic contents [8,12], and high concentrations of beta-carotene, niacin, and iron [13]. A complete nutritional profile has been produced by Maza-De la Quintana and Paucar-Menacho [14]. The health benefits also span consuming it as a raw fruit as well as in food products. Silva et al. [15] reported that lucuma extracts function as alpha-glucosidase inhibitors producing a hypoglycemic effect that may help in managing diabetes in patients. The high fiber composition of the fruit or pulp can lower the risk of diverticular diseases and metabolic syndrome [10]. A clinical study reported that *Pouteria lucuma* extract possesses potent anti-cancer properties [16]. Many lucuma species have shown commercial importance in food and traditional medicine [15]. The promising health benefits are making lucuma a vibrant ingredient, although it is still not fully explored in food products outside South American countries.

Sensory characteristics are essential for consumer acceptance of food products. Lucuma is reported to have a caramel, maple, pumpkin sweet flavor, floury texture, and intense yellow color [6]. Because of exceptional sensory characteristics, lucuma-flavored ice creams are extremely popular and often beat the more traditional vanilla and chocolate flavors in Peru and Chile. However, lucuma ice cream is not well-known outside of South America, and it is non-existent in the United States (US). This study investigated the sensory properties and acceptance of ice creams made with commercially available lucuma powders by US consumers.

Ice creams are extremely popular in the United States holding a significant market share. With growing health concerns among consumers, the demand for healthy alternatives in food products, including ice cream, has been increasing. New varieties of ice cream catering to the functional and dietary requirements of consumers are becoming popular. For example, companies have already made various attempts to provide healthier ice creams formulated with probiotics, prebiotics, dietary fibers [17], and amaranth [18]. The lucuma fruit is a good source of dietary fiber [10], delivers a sweet taste, and has a low glycemic index, which can provide an excellent functional ingredient for ice cream, smoothies, yogurt, cakes, cookies, etc. Studies suggest that, often, consumers perceive products formulated with healthy ingredients as less satisfying than non-healthy foods [19,20,21]. Thus, it is necessary to understand the US consumers’ attitudes towards lucuma ice cream, its acceptability, and perceived sensory characteristics [22] through a central location test (CLT).

A CLT is a common type of quantitative consumer test that is performed at a location organized by sensory professionals in a controlled environment [23]. Several research studies have used this approach to determine consumer liking or acceptance. De Souza Fernandes et al. [24] tested consumer acceptance and sensory properties of cassava-based ice creams. Friedeck et al. [25] used a CLT to collect consumer responses to protein fortification of ice cream. Cultural factors such as beliefs, meal preparation, meal preferences, and conditions of consumption can play a major role in food selection and can vary from one culture to another [26,27]. Considering the US consumers’ cultural unfamiliarity with lucuma ice cream, a food neophobia scale (FNS) developed by Pliner and Hobden [28] was used to measure consumer attitudinal aspects. Typically, in the FNS, respondents indicate their agreement or disagreement with 10 statements about foods or eating situations [29].

Overall, this research is the first of its kind that has attempted to study US consumers’ attitudes and liking of lucuma ice cream. The specific objectives of this study were (1) to investigate consumer liking of ice creams made with lucuma powder with different sensory profiles (purchased from different sources), (2) to understand how lucuma ice cream compares to a more mainstream caramel ice cream flavor that is more familiar to the US consumers, and (3) to assess food neophobia (FN) to segment the US consumers and understand how they differ on their perception of lucuma ice cream.

## 2. Materials and Methods

### 2.1. Selection and Preparation of Samples

Five out of twelve commercial lucuma powder samples were selected on the basis of aroma and volatile composition [30]. The list of ingredients and lucuma powder samples used in ice cream formulations are provided in Table 1.

The summary of odor descriptors presented in Table 2 is from earlier research by Singh [30] on volatile compounds and associated odors in twelve lucuma powder samples using a Gas Chromatography Olfactometry (GCO) analysis. Five of those lucuma powders are used in this study to prepare lucuma ice cream samples. The previous information generated by Singh [30] was used to select samples that were most different from each other to maximize differences and eliminate products that may have more similar characteristics. A more familiar caramel ice cream was used as a control product in comparison to the five lucuma-based ice cream samples. Ice creams were prepared using a standard base formulation at the Dairy Plant at Kansas State University (Manhattan, KS, USA). The formulation consisted of cream, milk, non-fat dry milk (NFDM), sugar, corn sugar, and stabilizer (icepro2004). All the ingredients except cream were mixed and heated to 38 °C. The cream was added to the mixture and pasteurized at 74 °C. The mixture was held at 74 °C for 30 min and then cooled down to 60 °C. Then, the mixture was homogenized at 8274 kPa (1200 psi) pressure. After homogenization, the mixture was cooled down to 10 °C and placed in a holding tank (Creamery Package Manufacturing Company, Lake Mills, WI, USA) for 24 h. Five percent of each lucuma powder and caramel were added to the base mix as flavoring agents. Each ice cream was prepared using a batch freezer (Emery Thompson Machine and Supply Co., Bronx, NY, USA). The final ice cream product consisted of approximately 12–13% fat (by weight), with approximately 11% total solids. Ice cream was transferred into half-gallon plastic containers and stored in a blast freezer overnight. The next day, all the ice cream samples were transferred to a walk-in freezer and stored at −18 °C until served to participants.

#### 2.1.1. Serving of Samples

One standard scoop (approximately 56 g) of each ice cream was served in 118 mL disposable polystyrene translucent plastic souffle cups (Dart, Mason, MI, USA) covered with clear lids. All sample cups were labeled with three-digit randomized codes. The consumers were presented with only one sample at a time with a 2 min break between each sample, and samples were referred to as “ice cream”. No flavor reference was provided at the time of serving. Table 3 shows the pictures of the samples used in the study. Ice cream samples were evaluated by participants in one session held in a day. Nabisco Premium unsalted crackers (Mondelez Global LLC, East Hanover, NJ, USA) and plain water were used as palate cleansers. General instructions were provided to participants on tasting the ice cream samples to form an opinion.

### 2.2. Participants and Procedure

#### 2.2.1. Participants

A total of 106 participants (males = 26, females = 80) were recruited from the Kansas City suburban area from a consumer database managed by the Sensory and Consumer Research Center at Kansas State University (Olathe, KS, USA). Participants aged between 18 and 65 years old, with no known food allergies or health problems, and who consumed ice cream at least once a month were prescreened for recruitment. Furthermore, participants were required to be frequent caramel ice cream eaters. Caramel ice cream was selected to be a part of this study as a comparative flavor because lucuma ice cream has been described as tasting “caramel-like”; therefore, it would provide a better comparison than any other ice cream flavor present in the US market. Also, participants should not have taken part in any consumer research in the past three months anywhere in the US [23]. Consumer demographics are shown in Table 4.

#### 2.2.2. Central Location Test

The CLT was conducted at the Sensory and Consumer Research Center, Kansas State University, Olathe, KS, USA. Compusense cloud (Compusense, Inc., Guelph, ON, Canada) was used for screening, recruitment, execution, and data collection. Participants signed an electronic informed consent and were compensated for their time. The samples were evaluated following a sequential monadic serving and were presented based on a completely randomized balance design generated in Compusense. The authors verified the design to ensure all samples were presented in all possible orders. The study was conducted under the existing Institutional Review Board (IRB) approval (#05930) using approved protocols.

### 2.3. Questionnaire

The questionnaire comprised various questions relating to the acceptability of the ice cream, sensory characteristics, and openness towards lucuma ice cream. Participants assessed the ratings for color, aroma, overall, flavor, texture, and aftertaste of each ice cream sample on a 9-point hedonic scale (“1 = dislike extremely”, “5 = neither like nor dislike” to “9 = like extremely”). An open-ended comment question for reasons of likes and dislikes was included to obtain consumer descriptors for each sample. Participants were asked to generate sensory profiling by rating the strength of specific attributes on a 7-point unstructured scale (1 = ‘none’ and 7 = ‘extreme’) [31]. The intensity ratings were asked for fruit flavor, caramel flavor, sweetness, and aftertaste. Additionally, a 5-point Just-About-Right (JAR) scale, ranging from 1 = “much too weak”, 3 = “just about right”, to 5 = “much too strong”, was used to determine product penalties by the consumers [32]. JAR questions were asked for fruit flavor, caramel flavor, sweetness, and powdery mouthfeel.

At the end of the questionnaire, participants were asked to agree or disagree with the 10 statements regarding FN on a 7-point scale (“1 = strongly disagree”, “4 = neither agree nor disagree”, to “7 = strongly agree”). Rabadán and Bernabéu [33] listed the statements used to examine consumer attitude and FN for trying unfamiliar or novel food products developed by Pliner and Hobden [28]. At the end of the questionnaire, participants were shown a concept consisting of a picture of lucuma fruit and a brief explanation regarding lucuma, its uses, and health benefits (Figure 1). After participants read through the lucuma concept, they were asked “how interested would you be in buying lucuma ice cream” on a 5-point scale (“1 = definitely will not buy, “3 = might or might not buy”, to “5 = definitely will buy”). All sensory and consumer evaluation results were collected between March and April 2021 using the Compusense cloud (Compusense, Inc., Guelph, ON, Canada).

### 2.4. Data Analyses

A one-way analysis of variance (ANOVA) using Tukey’s Least Significant Difference (LSD) was performed to identify significant differences (*p* < 0.05) between samples on liking and attribute intensity ratings. Penalty analysis was performed on JAR data to determine the penalties accrued by the participants [34]. Penalty analysis results also included the percent mean drop on the liking score when the participants did not rate a particular sample characteristic as JAR. Data analysis was performed using XLStat software version 2023.1.4 (Addinsoft, NY, USA). FN scores were calculated based on the sum of each response for a participant from the 10-item FNS. Then, food FN scores were divided into percentile cut-offs to classify the levels of FN as low, medium, and high. Five statements (1, 4, 6, 9, and 10) were reversed and considered while analyzing the results [33]. ANOVA test was performed for overall liking attribute to determine the differences between FN classifications.

## 3. Results

### 3.1. Demographic Information

The demographic information of the participants is presented in Table 4. The CLT was performed with 25% men and 75% women, and the age range was between 18 and 65 years. The qualified consumers consumed ice cream at least once a month.

### 3.2. Consumer Liking

The results of the ANOVA for consumer liking scores are shown in Table 5. The tested ice cream samples were significantly different for overall liking as well as aroma, flavor, and texture liking. The average overall liking score ranged from 4.75 (between “dislike slightly” and “neither liked nor dislike”) to 6.16 (between “like slightly” and “like moderately”). However, the mean overall liking scores for all lucuma ice cream samples ranged between “neither liked nor dislike” and “like slightly”. The mean overall liking score was highest for the Terrasoul sample followed by others such as Naturevibe, Superfood, Healthworks, and Herbazest. The caramel ice cream used as a control sample received the lowest mean overall liking. Terrasoul lucuma ice cream was also liked significantly more for aroma compared with other samples (*p* < 0.05). Terrasoul, Naturevibe, and Healthworks samples were liked significantly higher than the control sample (*p* < 0.05) for flavor. Surprisingly, the control sample was liked more for color and texture but was not significantly different from Terrasoul and Naturevibe. Consumers liked all samples equally for color, with mean scores above “liked slightly”. The aftertaste ratings for all samples were between “dislike slightly” and “neither liked nor dislike”, with no significant differences between samples. Aftertaste rating results suggest that the participants “neither liked nor disliked” the aftertaste of the samples. The overall liking, aroma, and flavor liking results suggest that consumers liked the lucuma-flavored ice cream over a regular caramel-flavored ice cream, especially the Terrasoul sample.

### 3.3. Attribute Intensity Rating

Participants evaluated the intensity of sensory attributes on a 7-point unstructured scale (1 = ‘none’ and 7 = ‘extreme’). The mean intensity ratings of three main sensory attributes rated by the consumers are shown in Table 6. The mean intensity rating scores ranged from 3.56 (lowest) to 4.55 (highest) across attributes, meaning that consumers perceived the ice cream samples as delivering medium intensities for caramel, sweetness, and overall aftertaste. The ice cream samples were found to be significantly different for caramel, sweetness, and aftertaste intensity. The Terrasoul sample was perceived to have a significantly higher mean intensity for caramel flavor, whereas the control sample was rated lower along with other samples. Similar to the caramel flavor trend, the Terrasoul sample was perceived as the most intensely sweet ice cream with the strongest aftertaste, followed by the control sample. The Herbazest sample was reported to have the weakest caramel flavor, sweetness, and aftertaste intensities.

### 3.4. Penalty Analysis Results

Penalty analysis explains the drop in liking due to less-than-optimal perception of various attributes such as color, fruit flavor, caramel flavor, sweetness, and powdery mouthfeel as measured by JAR scales. The percentage of consumer responses and mean drop values for corresponding attributes are shown in Table 7. Above 60% of consumers perceived the color as optimal or JAR for Healthworks, Herbazest, Naturevibe, and Terrasoul samples. Control and Superfood ice cream colors received less than 60% JAR scores. None of the ice cream samples saw a significant drop in liking scores for not having optimal color. All lucuma powder ice cream samples (Naturevibe, Terrasoul, Healthworks, Superfood, and Herbazest) were penalized (significant drop in liking mean) for having too little fruit flavor, caramel flavor, and sweetness. The control sample was also penalized for weak caramel flavor and sweetness.

Among the samples evaluated, Terrasoul ice cream received the highest JAR score for fruit flavor (35% of consumers), caramel flavor (54% of consumers), and sweetness (67% of consumers). Like JAR scores, the Terrasoul ice cream also received the highest intensity rating for caramel, sweetness, and aftertaste (Table 6). It appears that lucuma ice cream samples delivered slightly higher caramel flavor in comparison to fruit flavor. The mean drops were higher for fruit flavor, followed by caramel, sweetness, and powdery mouthfeel. As confirmed by attribute intensity ratings, the control ice cream sample was weakest on caramel flavor; penalty analysis showed that 60% of consumers saw it as having too little caramel flavor (Table 7). The control sample was not evaluated for fruit flavor on the JAR scale. Terrasoul and Healthworks were the only two samples seen by more than 60% of consumers as having appropriate sweet levels, although, it was still not enough to avoid a penalty.

Consumers noticed a strong (too much) powdery mouthfeel in lucuma ice cream samples. Perception of too much powdery texture could have negatively affected the liking scores and was responsible for a significant drop in mean liking. The control sample outperformed the lucuma ice creams in delivering appropriate mouthfeel and texture (67% of consumers). There was no significant drop in liking mean scores for powdery mouthfeel.

### 3.5. Food Neophobia Scale

**Figure 2:**
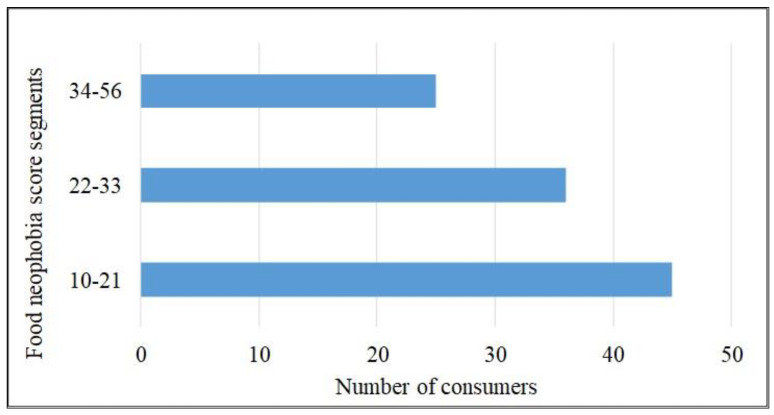
Food neophobia score segmentation using percentile cut-offs (*n* = 106 consumers).

The FN scores were calculated using participant responses to each of the statements on the FNS. The FN scores ranged from 10 to 56 (possible range of 10 to 70) with a mean score of 27.97 ± 11.63. The higher the FN score, the greater the degree of FN. Participants were classified by their degree of FN based on percentile cut-offs, including low range with scores from 10 to 21, medium range with scores from 22 to 33, and high range with scores from 34 to 56. The FN score segments are shown in Figure 2. Approximately 42% (45 out 106) of the participants had a lower range of FN scores, suggesting that they would have lower resistance to trying new foods, whereas 24% of the consumers would show very high reluctance towards new foods. Furthermore, close to 34% of the participants were moderately food neophobic [33].

Furthermore, the ANOVA results presented in Table 8 show that both the low group (FN score = 10–21) and the high group (FN score = 34–56) liked lucuma-formulated ice cream samples more than the control caramel (*p* < 0.05), whereas the medium group (FN score = 22–33) has lower liking scores for lucuma ice cream samples. This could be attributed to the fact that US consumers did not consider lucuma ice cream samples to be unfamiliar. Because lucuma has been reported to have a caramel-like flavor impression [6], and due to familiarity with caramel flavor, consumers might have shown a willingness to try lucuma-flavored ice cream despite having higher FN scores.

### 3.6. Purchase Intent Results

**Figure 3:**
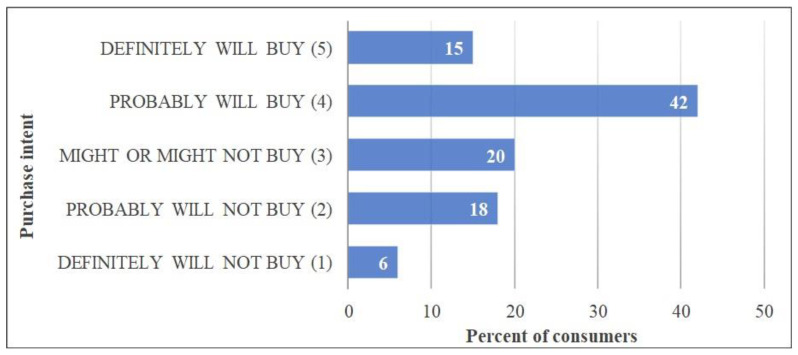
Consumers purchase intent scores after reading the lucuma concept.

Participants rated the purchase intent on a five-point scale, with the center point (3) as “might or might not buy”. The purchase intent question was not asked for each ice cream sample, rather it was intended to investigate the US consumers’ interest in lucuma-formulated ice cream as a novel concept. The participants were ice cream consumers, participating out of a desire to experience caramel ice cream. After the taste test, consumers were shown the lucuma fruit concept (Figure 1) and asked for their interest in buying lucuma ice cream. The histogram in Figure 3 displays the frequency distribution of scores corresponding to the scale used. A total of 15% of the consumers answered that they “definitely will buy” lucuma ice cream. A large share of participants i.e., 42%, responded that they “probably will buy”, whereas only 6% answered that they “definitely will not buy” a lucuma ice cream. Additionally, after linking the purchase intent data with FN segments, it was found that 64% of consumers in the high food neophobic score (34–56) group indicated that they would “probably or definitely” buy lucuma ice cream, whereas only 20% of the participants responded by saying that they would “probably or definitely not” buy a lucuma ice cream. These results are contrary to the idea that higher food neophobic consumers would be less willing to try new or unfamiliar products. Consumer perception might have changed based on the number of health benefits mentioned in the concept.

## 4. Discussion

To date, this is the first study to have developed and tested lucuma-formulated ice creams among US consumers. This study established the fact that commercially available lucuma powders in the US market vary widely in their perceived sensory experience when formulated in an ice cream recipe. The ice cream samples covered a wide range of all liking modality ratings, confirming that a broad sensory range of products was developed and evaluated.

### 4.1. Comparison of Hedonics among Ice Cream Samples

The ice cream samples had the same base recipe, differing only in the type of lucuma powder. The amount of lucuma powder was kept the same across all ice cream recipes, while the control sample did not have lucuma powder but did have caramel flavor in the formulation. The mean overall liking scores of the ice cream samples indicated that consumers either “liked slightly” or “neither liked nor disliked” the samples evaluated, with the exception of the control sample, which received lower liking. The overall liking was strongly affected by aroma, flavor, texture, and aftertaste liking, and, to a lesser extent, appearance liking. The Terrasoul sample was the most liked overall, and it also received significantly higher aroma and flavor liking ratings. Naturevibe was not significantly different from Terrasoul for overall and flavor liking. A possible reason could be the strong intensity and distinct aroma and flavor characteristics of Terrasoul lucuma powder. In Table 2, GCO results confirm the presence of strong buttery, sweet, caramel, and brown sweet aromatics in Terrasoul powder [30]. The combination of buttery, caramel, and sweet aromas could have positively affected consumed appreciation of the Terrasoul ice cream sample. The top three frequent descriptors consumers used to describe the Terrasoul sample were creamy, caramel, and sweet (Table 9). According to Bullock et al. [35] and Cadena et al. [36], the combination of creamy and sweet attributes is a key driver of liking for ice creams.

Naturevibe can be referred to as the second most liked sample, although it was not significantly different from Herbazest, Superfood, and Healthworks. Naturevibe powder was reported to have compounds responsible for brown sweet and fruity aromatics, which can increase the overall impression of positive drivers of liking (Table 2). Consumers described it as creamy, sweet, caramel, and smooth ice cream (Table 9). No significant difference was found between Herbazest, Superfood, and Healthworks for overall, aroma, and flavor liking scores. The overlap in hedonic results for the above-mentioned samples is explained by poor differentiation of those samples on color, aroma, flavor, and aftertaste. A predominant powdery texture could have been another reason for consumer dislike (Table 9). Results of penalty analysis (Table 7) and attribute intensity rating (Table 6) attest that consumers perceived all lucuma ice cream samples as mild in flavor (sweet, fruit, and caramel), perhaps hindering the acceptance of those samples. Moreover, the unfamiliarity of consumers with lucuma ice creams could have also been a determining factor for both liking and differentiation. Many studies reported that food and beverage familiarity related positively with product liking and sensory differentiation [37]. For example, fruit, wine, chocolate bars, kiwifruit [38], cheese [39], soy sauce [40], ready-to-eat meals [41], and red wine [42] reported higher ratings due to familiarity.

The control sample was liked significantly less overall as well as for flavor. The absence of lucuma powder in the control sample may have significantly affected overall flavor perception, and caramel and fruity flavors. A compounding effect of weak flavors is likely the main reason for consumer rejection of the control sample. Consumers commented about control samples being creamy, smooth, and sweet, but very few mentioned caramel flavor (Table 9). It is important to note that the control sample did include caramel flavor in the formulation, but the amount may have been too low for consumers to perceive. Nevertheless, among all samples, the control was successful in delivering a more satisfactory texture experience. It received numerically the highest texture liking (Table 5), and 67% of consumers perceived it as optimal (JAR) for texture mouthfeel, meaning not having too much powdery mouthfeel (Table 7). The control sample is the only sample that did not receive a penalty for too much powdery texture, which confirms the presence of a creamier texture experience. None of the consumers mentioned powdery, chalky, gritty, or grainy for the control sample (Table 9). The impact of lucuma powder inclusion on texture is evident in penalty analysis (Table 7) and consumer descriptors (Table 9) where consumers noticeably reported high powdery, chalky, gritty, and grainy mouthfeel. This could have been one of the main reasons for the lucuma ice cream samples getting overall low mean liking scores. Other studies have also described creamy texture mouthfeel (free from gritty, grainy, chalky, and powdery residue) as the most desired modality after flavor for ice creams [36,43,44,45].

The hedonic results indicate that consumers liked the ice cream samples to some degree. Ice creams formulated with Terrasoul and Naturevibe were liked significantly more than other lucuma variants, including the caramel control. The liking results provide evidence that lucuma-flavored ice creams are accepted by US consumers. Further research would be needed to find ways to maximize flavor and texture acceptance. Also, lucuma powder variants such as Terrasoul and Naturevibe are likely to receive higher consumer acceptance over Herbazest, Superfood, and Healthworks variants. The various lucuma powders affected sensory modalities (aroma, flavor, and texture) differently. Further improvements, for example, strengthening flavor intensities (caramel, fruit, sweet) and reducing powdery texture are needed to increase consumer liking.

### 4.2. Comparison of Sensory Attribute Intensity Ratings

Participants rated caramel flavor, sweetness flavor, and aftertaste intensity on a 7-point unstructured scale (1 = ‘none’ and 7 = ‘extreme’). The ice cream samples differed significantly on all three attributes (Table 6). Terrasoul lucuma ice cream delivered the highest caramel flavor, followed by numerically higher sweetness intensity. Other ice cream samples did not significantly differ in caramel, sweetness, and aftertaste intensities. The Herbazest sample was perceived as having the weakest intensity based on all three measured attributes. The control ice cream was one of the lowest-rated samples for caramel flavor intensity. This could be attributed to the percentage of caramel flavor used while preparing the ice cream. Perhaps there was more space to add a higher percentage of caramel flavor to the base recipe, but it was kept at the same level as lucuma powder percentages to maintain consistency across the samples. Both Terrasoul and control samples were perceived to have significantly higher sweetness and aftertaste intensities (*p* < 0.05) than the rest of the samples. Overall, the equally added quantities of lucuma powder variants were not successful in meeting consumer expectations for caramel and sweetness in ice cream samples, except for Terrasoul. Consumers termed all ice cream samples as creamy, but Terrasoul and Naturevibe were rated higher for delivering caramel and sweet flavor; the control was referenced least for caramel flavor, confirming that lucuma can fulfill the expectation of caramel flavor (Table 9).

The attribute rating evaluation was performed by untrained participants. Therefore, if there were lower magnitude differences between samples, it may have been overlooked by the untrained participants. Another potential reason for the low differentiation could be the unfamiliarity of consumers with the novel taste of lucuma ice cream, which can hinder their ability to perceive and differentiate low–medium intensity flavor attributes. Food developers can design future studies using different quantities of lucuma powder variants coupled with stabilizers and emulsifiers to improve the texture of ice cream. The scope of this study was limited to measuring consumer acceptance of lucuma ice cream, but future studies can also utilize trained descriptive sensory panels to determine and quantify the differences between key sensory attributes even at lower magnitudes.

### 4.3. Penalty Analysis

Penalty analysis results indicate that the inclusion of lucuma powder improved the yellow color of Terrasoul, Healthworks, Herbazest, and Naturevibe samples in comparison to the control sample, except for the Superfood variant. More than 63% of consumers felt that the yellow color of these ice cream samples was close to just about right. This fact implies that the respective lucuma powder types (Terrasoul, Healthworks, Herbazest, and Naturevibe) do have the potential to contribute positively to ice cream color. The control sample color was much too light yellow, and the Superfoods ice cream color was inconsistent, as consumers were split between too light and too dark. Previous research work also reported intense yellow color as a key appearance property of lucuma fruit powder [6]. The lucuma powder varieties differ in fruit and caramel flavor intensity as reflected in the penalty analysis. Among samples, the ice cream formulated with Terrasoul was successful in terms of delivering the highest consumer responses to JAR ratings for fruit flavor, caramel flavor, and sweetness, but was still not enough to lower the penalty. Studies by Yahia and Guttierrez-Orozco [6] and Ren et al. [46] found caramel, tropical fruit, sweet flavor, and yellow color in lucuma fruit powders. Except for the control, all ice cream samples were penalized for excessive powdery mouthfeel and consumer descriptors also reinforced this finding (Table 9). A clear indication that the addition of lucuma powder contributed to the chalky and powdery texture that resulted in the disruption of the creamy texture of the ice cream [6].

Although some of the samples had lower mean drops, they still received a higher penalty because of the high percentage of consumers who rated the products away from JAR. The Naturevibe sample is an example of such a penalty. Terrasoul was the most liked sample overall and also received mean drops of greater than two points for having too little caramel flavor and sweetness. A similar trend of decline in overall liking for weak fruit and caramel flavor was seen for Healthworks. Superfood by MRM and Herbazest samples also received higher mean drops for low caramel flavor.

Even though there is evidence of various lucuma powders affecting the flavor and texture attributes differently, consumers still did not like them for having too little fruit flavor, caramel flavor, and sweetness, as well as for powdery mouthfeel. Strong penalties across the samples suggest that all the attributes studied on the JAR scale matter to the consumers, as they rated the products lower when the attributes were perceived to not be JAR. The findings conclude that the consumer liking scores of lucuma ice cream can be further improved if gaps in flavor intensities are filled. Adding more lucuma powder to the ice cream recipe may not offer an appealing texture and could contribute towards a powdery and chalky texture. Improvement in ice cream texture is as vital as enhancing the flavors of lucuma ice creams. Another alternative could be to use other forms of lucuma such as pulp, which could increase costs. Other studies have also suggested that enhancement in caramelized flavor and creamy texture [44], fruit flavors [43], a combination of taste and flavors [35], color, creamy texture, and sweet flavor [36] contribute positively to the acceptance of ice cream.

### 4.4. Food Neophobia

Due to unavailability, people living in the US rarely consume lucuma-formulated ice cream. This study aimed to understand how US consumers would react to lucuma-formulated ice cream. All participants completed the FNS questionnaire after evaluating lucuma ice cream samples. They were asked to rate the level to which they agreed or disagreed with FNS statements on a 7-point scale. An FN score of 34 or higher is considered high neophobia, between 22 and 33 is moderate, and less than 21 is low neophobia [47]. Overall, the participants in this study had an average score of 27.97 ± 11.63, indicating that the US consumers had low–moderate FN. The ice cream sample tasting allowed us to set the context, therefore improving the reliability of the results. The analysis of the FN score based on participants’ demographics and ice cream consumption incidence rate is presented in Table 4. This study found that male participants had lower scores than females. In a previous study conducted at Bournemouth University, male students had consistently lower scores than females [48]. Additionally, participants aged between 45 and 65 years old had lower FN scores than the overall mean score. There is also a trend of a decline in FN scores with an increase in participants’ age, starting from 35–44 years old to 55–65 years old. Typically, humans reach the highest level of FN during childhood, but FN declines in adolescence, remains stable during adulthood, and increases again in old age due to health concerns and sensory sensitivity [49].

The participants with extreme ice cream consumption frequency (daily and at least once a month) had very low neophobia scores; however, both these consumer groups account for only 10% of the total participants. The results clearly assert that US consumers are not abhorrent to the idea of lucuma-formulated ice cream, and improvements to flavor and texture experience with proper messaging can further reduce FN scores. Food developers and companies should consider introducing lucuma-powdered products into the US market to reduce the reluctance for lucuma food products. Barton et al. [50] showed that consumers’ original unwillingness disappears once the food is no longer novel. Similarly, Predieri et al. [51] concluded that greater exposure to food and cultural diversity can minimize FN scores.

### 4.5. Purchase Intent

Overall, 57% of consumers had a purchase intent of 4 or higher, indicating “probably will buy” and “definitely will buy”, despite scoring around the mid-point “neither liked not disliked” on a 9-point liking scale. A possible reason for the high purchase intent could be the health benefits mentioned along with the lucuma fruit picture. The information presented in the concept seems to have possibly affected consumers’ judgment on purchase intent. Other studies have confirmed this phenomenon, that the health benefits of products positively influence consumers’ purchase interest [52,53]. Furthermore, the curiosity factor of trying something new originating in a foreign country, and the idea of trying it in an ice cream could also have contributed positively [54]. Perhaps the emotion of novelty was strong enough that it led to 77% of consumers choosing neutral to positive purchase intent “might or might not buy”, “probably will buy”, and “definitely will buy” toward lucuma ice cream.

By analyzing purchase intent results with FN segmentation, almost 64% of the high (FN score = 34–56) group chose that they would “probably or definitely” buy lucuma ice cream, and only 20% of the participants responded that they would “probably or definitely not” buy lucuma ice cream. These findings are contrary to the usual notion that higher food neophobic consumers would be less willing to try new or unfamiliar products. As explained earlier, consumers’ perceptions might have changed based on the number of health benefits mentioned in the concept, the interest in trying a new exotic fruit ice cream, and the fact that it has an acceptable flavor to consumers. Possibly, if the lucuma fruit concept had been presented and disclosed before the tasting, consumers might have rated the ice cream samples higher knowing the health benefits of lucuma. Further research will be needed to confirm this hypothesis.

## 5. Conclusions

The objective of this study was not to develop an ideal formulation but rather to assess a novel lucuma ice cream concept potential in the US market. Results showed that lucuma and control ice cream samples were rated differently by consumers. Consumer ratings for overall liking, aroma, and flavor were significantly higher (*p* < 0.05) for the lucuma-powder-formulated ice cream samples, whereas texture liking was significantly higher (*p* < 0.05) for the control sample. The Terrasoul variant was the most liked sample, successful in delivering relatively higher caramel, brown, buttery, and sweet flavors, even though consumers perceived all ice cream samples as weak in flavor. The inclusion of lucuma powder enhanced the caramel and sweet aroma intensities, which positively affected ice cream acceptance. The addition of lucuma powder increased the powdery and chalky mouthfeel while reducing the creamy texture experience; this negatively affected consumer acceptance.

Consumers with lower FN scores showed a willingness to try new or unfamiliar foods. Forty-two percent of consumers with low FN scores responded that they would probably buy lucuma ice cream, if available on the shelves. The study findings help us to understand the US consumers’ perception of lucuma ice cream and show a potential market space for it in the US, especially if positioned as a healthier alternative. Future research can explore ideal formulations of lucuma ice cream and can also determine the possibilities of using lucuma fruit in other commercial products such as yogurts, smoothies, and baked goods.

The ice creams developed in this project were not developed to compete against a commercial in-market product. Thus, there is a possibility of a further increase in consumer liking by product refinement. This work focused on the taste perception of lucuma ice cream samples to understand consumers’ willingness to consume new flavors. Future research is required to learn the effects of other variables such as cultural background, dietary habits, economic income, etc., on willingness to consume novel flavors. Additionally, the amount of caramel and lucuma powder used in ice cream samples for flavorings was kept equal to maintain formulation consistency. In hindsight, perhaps the percentage of caramel in the sample could have been higher, as consumers noticed it was not as intense as lucuma powder ice creams, which eventually made consumers describe it as bland. This study provides the perception of consumers from the Kansas City area. It would be interesting to examine how lucuma ice cream would perform if tested in other locations throughout the US that may have access to other flavors and to gather more insights from a larger population base.

## Figures and Tables

**Table 1:** List of ingredients used in the formulation of ice cream samples.

Ingredients	Description
Whole milk	Pasteurized whole milk (3.25% fat), Great value, Walmart, Bentonville, AR
Heavy cream	Pasteurized frozen heavy cream (36% fat), Hiland, Kansas City, MO
Non-fat dry milk	Grade A non-fat dry milk, Dairy America, Fresno, CA
Sucrose	Sugar, granulated, extra fine cane, Sysco, Houston, TX
Stabilizers/emulsifiers	Grindsted icepro, Danisco, New Century, KS
Soluble corn solids	Maltrin m200, Grain processing corporation, Muscatine, IA
Caramel flavor	If applicable
Lucuma fruit powder varieties	
Healthworks	Healthworks, Scottsdale, AZ
Herbazest	Herbazest Inc., Orlando, FL
Naturevibe	Naturevibe Botanicals, Rahway, NJ
Superfoods	MRM Nutrition, Oceanside, CA
Terrasoul	Terrasoul Superfoods, LLC., Fort Worth, TX

Note: The samples were prepared using a base formulation of commercial ice cream, a trademark product of the Dairy Plant at Kansas State University (Manhattan, KS, USA); thus, the exact formulation values cannot be shared due to a confidentiality agreement.

**Table 2:** Summary of odor descriptors based on the concentrations of active volatile aroma compounds found in six lucuma power varieties. The descriptors are listed from highest (top) to lowest (bottom) concentration of volatile compounds.

Naturevibe	Terrasoul	Healthworks	Superfood	Herbazest
Plastic, Solvent, Chemical	Grain, Musty, Leather	Burnt, Plastic, Musty/dusty, Chemical, Sour	Sweet, Sour, Chemical	Buttery, Sweet
Brown sweet, Sour	Buttery	Grain, Chemical	Green	Burnt, Leather, Grain
Cucumber	Alcohol, Sour	Green	Burnt, Grain, Waxy	Baked potato, Grain
Plastic, Mushroom, Waxy	Green	Buttery	Baked potato	Cucumber
Baked Potato	Baked potato	Musty/dusty, Musty, Plastic	Grain, Musty/dusty, Leather	Plastic, Mushroom
Green	Sweet, Waxy	Cucumber	Cucumber	Leather, Burnt, Caramelized
Burnt, Leather, Musty/dusty	Cucumber	Baked potato, Musty/dusty, Sweet	Mushroom, Plastic, Sweet	Nutty, Grain
Grain, Burnt, Caramelized, Musty dusty	Sweet, Caramelized	Musty/dusty, Mushroom	Burnt, Waxy, Plastic	
Plastic, Burnt, Waxy, Musty/dusty	Leather, Burnt	Burnt, Smoke	Burnt, Hay-like	
Sweet, Fruity, Musty/dusty, Fruity fermented	Brown sweet, Sweet	Leather, Smoke		
Leather, Musty/dusty, Nutty	Burnt, Plastic, Waxy	Sweet, Burnt		
Brown sweet, Sweet	Grain, Nutty, Musty/dusty	Leather, Waxy, Plastic		
Waxy, Plastic, Leather	Musty/dusty, Burnt	grain, Nutty		
Grain, Nutty				
Nutty, Spicy				

Note: This table is produced from the earlier research by Singh [30].

**Table 3:** Ice cream samples used in the consumer test.

Sample Name	Picture	Sample Name	Picture
Naturevibe	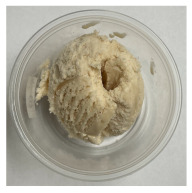	Healthworks	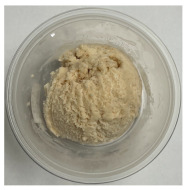
Terrasoul	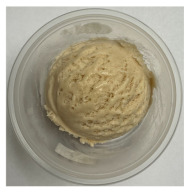	Herbazest	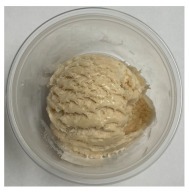
Superfood	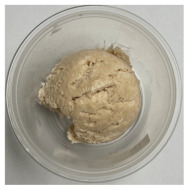	Control	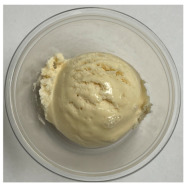

**Table 4:** Demographic profiles, mean scores, and standard deviations of the food neophobia score for each participant factor.

Category	Subcategories	Percentage (*n* = 106)	Food Neophobia Score (Mean ± SD)
Gender	Female	75%	27.97 ± 11.82
	Male	25%	25.0 ± 8.72
Age range	18–24 years	2%	27.5 ± 4.95
	25–34 years	10%	23.11 ± 9.39
	35–44 years	26%	27.74 ± 11.19
	45–54 years	22%	23.56 ± 12.76
	55–65 years	40%	26.68 ± 9.12
Ice cream consumption	Daily	4%	20.5 ± 11.21
	Once a week	26%	27.15 ± 9.85
	Several times a week	52%	27.54 ± 10.94
	2–3 times a month	12%	29.27 ± 10.59
	At least once a month	6%	22.33 ± 8.36

**Table 5:** Consumer hedonic ratings of ice cream samples measured on a 9-point hedonic scale.

	Control	Healthworks	Herbazest	Naturevibe	Superfood	Terrasoul	*p*-Value
Overall liking	4.75 c	5.44 b	5.38 b	5.78 ab	5.53 b	6.16 a	<0.0001
Color liking	6.52	6.17	6.18	6.20	5.97	6.20	0.268
Aroma liking	5.32 b	5.31 b	5.22 b	5.47 b	5.30 b	5.89 a	0.000
Flavor liking	4.43 c	5.53 ab	5.34 b	5.61 ab	5.35 b	6.09 a	<0.0001
Texture liking	6.16 a	5.06 c	5.55 bc	5.85 ab	5.50 bc	5.82 ab	0.006
Aftertaste liking	4.71	4.64	5.05	5.02	4.86	5.22	0.273

Note: Mean values within a row with the same letter are not significantly different at *p* < 0.05. Pairwise comparisons are only reported for the attributes that showed a significant *p*-value.

**Table 6:** Sensory attribute intensity ratings of key flavor attributes on a 7-point scale.

	Control	Healthworks	Herbazest	Naturevibe	Superfood	Terrasoul	*p*-Value
Caramel flavor	3.59 b	3.69 b	3.57 b	3.84 b	3.64 b	4.55 a	<0.0001
Sweetness	3.97 ab	3.93 abc	3.60 c	3.84 bc	3.76 bc	4.25 a	0.016
Aftertaste	4.38 a	4.01 ab	3.74 b	4.04 ab	3.99 ab	4.35 a	0.023

Note: Mean values within a row with the same letter are not significantly different at *p* < 0.05. Pairwise comparisons are only reported for the attributes that showed a significant *p*-value.

**Table 7:** Penalty analysis results showing the percentage of consumer responses and mean drop scores for color, fruit flavor, caramel flavor, sweetness, and powdery mouthfeel on the Just-About-Right scale.

	JAR ^3^ Level	Color	Mean Drop ^1^	Fruit Flavor ^2^	Mean Drop	Caramel Flavor ^2^	Mean Drop	Sweetness ^2^	Mean Drop	Powdery Mouthfeel ^2^	Mean Drop
Healthworks	Too little	23%	1.21	68%	2.04 *	49%	2.40 *	25%	2.00 *	6%	
	JAR ^3^	69%		28%		42%		60%		37%	
	Too much	8%		4%		9%		14%		58%	1.04 *
Herbazest	Too little	21%	0.24	64%	1.94 *	55%	2.24 *	35%	1.97 *	8%	
	JAR ^3^	64%		26%		36%		59%		43%	
	Too much	15%		9%		9%		6%		49%	1.44 *
Naturevibe	Too little	25%	0.50	71%	1.27 *	54%	1.75 *	31%	1.73 *	8%	
	JAR ^3^	63%		27%		36%		59%		47%	
	Too much	11%		2%		10%		9%		45%	1.05 *
Superfood	Too little	25%	0.51	71%	1.04 *	55%	2.05 *	33%	1.72 *	4%	
	JAR ^3^	53%		25%		37%		58%		47%	
	Too much	22%	0.67	5%		8%		9%		49%	1.24 *
Terrasoul	Too little	12%		58%	1.35 *	33%	2.08 *	22%	2.13 *	3%	
	JAR ^3^	73%		35%		54%		67%		47%	
	Too much	15%		7%		13%		11%		50%	1.64 *
Control	Too little	33%	0.12	na	-	60%	2.55 *	34%	1.86 *	14%	1.27
	JAR ^3^	59%		na	-	22%		47%		67%	
	Too much	8%		na	-	18%		19%		19%	1.82

^1^ The mean drops in the “too much” and “too little” levels are the differences between the liking mean for the JAR levels minus the “too much” or “too little” levels. Only mean drops for >20% of participants are reported. ^2^ Percentage of consumers with the respective attribute. ^3^ JAR: Just about right. * Significant mean drop score at *p* < 0.05. - There was no fruit flavor in the Control samples; thus, it was not measured.

**Table 8:** Comparison of analysis of variance results of overall liking scores for low, medium, and high food neophobia score groups.

	Low FN Score (10–21)	Medium FN Score (22–33)	High FN Score (34–56)
Terrasoul	6.47 a	5.81	6.20
Naturevibe	5.82 ab	5.47	6.08
Healthworks	5.67 ab	5.42	5.72
Superfood	5.60 ab	5.33	5.48
Herbazest	5.51 ab	4.97	5.16
Control	4.71 b	4.86	4.64
*p*-value	0.006	0.385	0.049

Note: Mean values within a column with the same letter are not significantly different at *p* < 0.05. Pairwise comparisons are only reported for the attributes that showed a significant *p*-value.

**Table 9:** Sensory characteristics used by consumers to describe perceived attributes of each ice cream sample. The attributes are listed from most (left) to least (right) frequent. Attributes mentioned at least three times were included.

Samples	List of Consumer Descriptors
Control	Creamy, Smooth, Sweet, Artificial, Bland, Caramel, Sour
Healthworks	Creamy, Powdery, Sweet, Caramel, Smooth, Grainy, Gritty, Bland, Chalky, Buttery
Herbazest	Creamy, Powdery, Chalky, Sweet, Smooth, Caramel, Bland, Gritty, Grainy, Fruity
Naturevibe	Creamy, Sweet, Caramel, Smooth, Chalky, Powdery, Gritty, Bland, Grainy, Sour
Superfood	Creamy, Powdery, Sweet, Caramel, Smooth, Bland, Artificial, Grainy, Chalky, Gritty
Terrasoul	Creamy, Caramel, Sweet, Powdery, Smooth, Chalky, Gritty, Nutty, Grainy, Artificial

## Data Availability

The raw data supporting the conclusions of this article will be made available by the authors upon request.
